# A Comprehensive Review of the Protein Subunit Vaccines Against COVID-19

**DOI:** 10.3389/fmicb.2022.927306

**Published:** 2022-07-14

**Authors:** Mohsen Heidary, Vahab Hassan Kaviar, Maryam Shirani, Roya Ghanavati, Moloudsadat Motahar, Mohammad Sholeh, Hossein Ghahramanpour, Saeed Khoshnood

**Affiliations:** ^1^Cellular and Molecular Research Center, Sabzevar University of Medical Sciences, Sabzevar, Iran; ^2^Clinical Microbiology Research Center, Ilam University of Medical Sciences, Ilam, Iran; ^3^Toxicology Research Center, Medical Basic Sciences Research Institute, Ahvaz Jundishapur University of Medical Sciences, Ahvaz, Iran; ^4^School of Paramedical Sciences, Behbahan Faculty of Medical Sciences, Behbahan, Iran; ^5^Department of Microbiology, School of Medicine, Ahvaz Jundishapur University of Medical Sciences, Ahvaz, Iran; ^6^Department of Microbiology, Pasteur Institute of Iran, Tehran, Iran; ^7^Faculty of Medical Sciences, Tarbiat Modares University, Tehran, Iran

**Keywords:** SARS-CoV-2, vaccine, COVID-19, protein subunit, review

## Abstract

Two years after severe acute respiratory syndrome coronavirus-2 (SARS-CoV-2), in December 2019, the first infections were identified in Wuhan city of China. SARS-CoV-2 infection caused a global pandemic and accordingly, 5.41 million deaths worldwide. Hence, developing a safe and efficient vaccine for coronavirus disease 2019 (COVID-19) seems to be an urgent need. Attempts to produce efficient vaccines inexhaustibly are ongoing. At present time, according to the COVID-19 vaccine tracker and landscape provided by World Health Organization (WHO), there are 161 vaccine candidates in different clinical phases all over the world. In between, protein subunit vaccines are types of vaccines that contain a viral protein like spike protein or its segment as the antigen assumed to elicit humoral and cellular immunity and good protective effects. Previously, this technology of vaccine manufacturing was used in a recombinant influenza vaccine (RIV4). In the present work, we review protein subunit vaccines passing their phase 3 and 4 clinical trials, population participated in these trials, vaccines manufactures, vaccines efficiency and their side effects, and other features of these vaccines.

## Introduction

The world has been fighting coronavirus disease 2019 (COVID-19) illness for nearly 2 years (Koupaei et al., 2022a; Naimi et al., 2022). COVID-19 was induced by severe acute respiratory syndrome coronavirus 2 (SARS-COV-2), which emerged in Wuhan, China (Koupaei et al., 2021; Mahdizade Ari et al., 2022). Traditional vaccine strategies developed on the basis on attenuated or inactivated pathogens have been demonstrated to be highly efficient for several infectious diseases (Khoshnood et al., 2022). However, in some situations, the whole pathogen method cannot provide the needed protection without adverse reactions viz inflammation, allergic, and autoimmune responses (Baird and Lopata, 2014).

Subunit vaccines based on microorganisms’ fragments have the ability to overcome these challenges. Subunit vaccines contain only include the pathogens antigenic components that are required to elicit effective immune responses. A polysaccharide, a nucleic acid or a protein can all be used as antigen (Bill, 2015).

**FIGURE 1:**
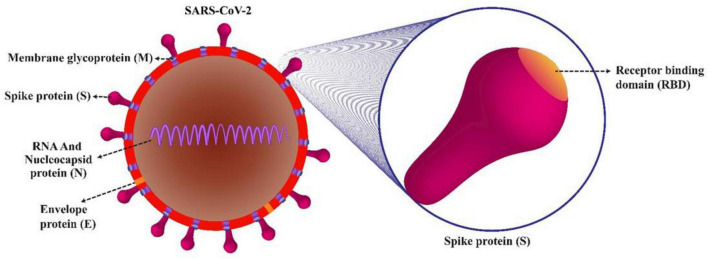
Structural proteins of SARS-CoV-2 virion. Nucleocapsid (N) protein is associated to the genomic RNA and S glycoprotein/spike (S), membrane (M), and envelope (E) proteins, which are located in the viral phospholipid bilayer. Protein S consists of two subunits, S1 and S2. The S1 subunit is a C-terminal receptor-binding domain (RBD) that detects the receptor, and the S2 subunit is used for membrane fusion, which is required to enter the host cell.

Protein subunit that contains a specific product of the virus rather than complete viral particle is used to elicit immune responses. In addition to accessory proteins, SARS-CoV-2 comprises of structural and non-structural proteins. S, membrane (M), and envelope (E) proteins are the main structural proteins of SARS-CoV-2 (Koupaei et al., 2022b). These proteins are situated in the viral phospholipid bilayer and in the nucleocapsid (N) protein, the ribonucleoprotein core (Figure 1). The S-proteins by binding to ACE2 (angiotensin-converting enzyme-2), facilitate host cell attachment and viral entry. Protein S consists of two subunits, S1 and S2. The S1 subunit is a C-terminal receptor-binding domain (RBD) that detects the receptor, and the S2 subunit is used for membrane fusion, which is required to enter the host cell. M proteins play a role in the formation of virion envelope. Antibodies neutralize the virus by binding to the S protein, thus making the spike gene sequence or protein a key component of SARS-CoV-2 vaccines. E proteins are important for SARS-CoV-2 infectivity and N-protein binds the viral RNA genome and forms the helical (Currier et al., 2021).

**FIGURE 2:**
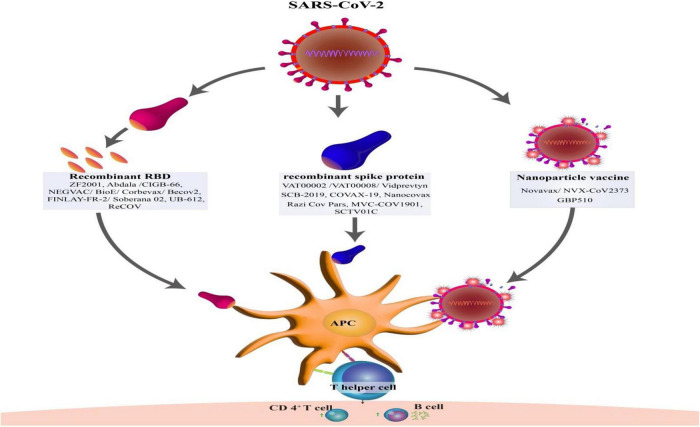
Type of structures protein subunit vaccines against SARS-CoV-2 and development of T-cell and B-cell immunity by activation of antigen-presenting cells (APCs).

S protein of SARS-CoV-2 has been shown, to be an ideal target for vaccine development on multiple platforms due to its high antigenicity and potency to induce robust immune responses (Figure 2; Martínez-Flores et al., 2021). However, since only a few viral components are included in the protein subunit vaccine that do not exhibit the full complexity of the virus antigen, their protective effect may be limited and, in some cases, may elicit unbalanced immune responses (Dong et al., 2020).

This article reviews the protein subunit SARS-CoV-2 vaccine approaches (Table 1) with a special focus on the efforts currently underway in the development of protein subunit SARS-CoV-2 vaccines. Here, we describe the all studies of the protein subunit SARS-CoV-2 vaccine and demonstrate their immunogenicity, efficacy, and safety.

**TABLE 1:** Protein-based vaccine against COVID-19 in clinical trials.

Vaccine name	Developer	Route of administration/ Dose	Clinical stage	Type of subunit and structure	Type of adjuvant	Efficacy	Side effects	References
**Recombinant spike protein vaccines**								
VAT00002/ VAT00008/ Vidprevtyn	Sanofi Pasteur (French) and GSK (United Kingdom)	IM/2	Phase 3 (Ongoing)	Recombinant spike protein [1. monovalent vaccine comprising spike protein D614 variant 2. bivalent vaccine comprising spike protein of D614 and Beta variant (B.1.351)]	AS03	NR	NR	Kandimalla et al., 2021
SCB-2019	CloverBiopharmaceuticals Inc. (China)/GSK)/Dynavax (United States)	IM/2	Phase 3 (data reported)	Recombinant trimeric spike protein	AS03 (GSK) or CpG/Alum	67.2% overall efficacy against any severity, 83.7% against moderateto-severe and 100% against severe COVID-19	Pain at the injection site, headache, fatigue, fever and myalgia	Richmond P. et al., 2021; Bravo et al., 2022
COVAX-19	Vaxine Pty Ltd. (Australia)	IM/2	Phase 3 (ongoing)	Recombinant spike protein	Advax-SM	NR	NR	Chavda et al., 2021
Nanocovax	Nanogen Pharmaceutical Biotechnology JSC. in (Vietnam)	IM/2	Phase 3 (ongoing)	Recombinant spike protein	Al(OH)3	NR	Pain at the injection site, fatigue, fever, headache, cough, sore throat in some volunteers and one patient exhibited serious sepsis	Hosier et al., 2020; Jacob et al., 2020
Razi Cov Pars	Razi Vaccine and Serum Research Institute (Iran)	IM/2 and IN/1	Phase 3 (ongoing)	Recombinant spike protein	NR	NR	Headache, mild fever and injection site pain	Pilicheva and Boyuklieva, 2021
MVC-COV1901	Medigen Vaccine Biologics Corporation (Taiwan)/NIAID/Dynavax (United States)	IM/2	Phase 3 (ongoing)	Recombinant spike protein (S-2P)	CpG 1018 and Al(OH)3	NR	Pain at the injection site, malaise, fatigue and fever was rarely reported (based on phase 2).	Hsieh et al., 2021
EPIVACCORONA VACCINE (EVCV)	Vektor State Research Center of Virology and Biotechnology, Koltsovo, (Russia)	IM/2	Phase 3 (ongoing)	Chemically synthesized peptide immunogens of the S protein of SARS-CoV-2 conjugated to a carrier protein	Al(OH)3	79%	Local pain at the injection site	Ryzhikov A. et al., 2021
SCTV01C	Sinocelltech Ltd.	IM/2	Phase 2/3 (ongoing)	Recombinant bivalent trimeric S protein	SCT-VA02B	NR	NR	Eroglu et al., 2021
**Recombinant RBD vaccines**								
ZF2001	Anhui Zhifei Longcom Biopharmaceutical/Institute of Microbiology, Chinese Academy of Sciences (China)	IM/2 or 3	Phase 3 (ongoing)	Tandem-repeat dimeric RBD protein	Al(OH)3	NR	Injection-site pain, redness, swelling at injection site and low systemic adverse reactions such as fever, fatigue, headache, nausea, Cough and muscle pain (based on phase 2)	Yang et al., 2021
Abdala/CIGB-66	Center for Genetic Engineering and Biotechnology (CIGB)	IM/3	Phase 3 (ongoing)	Monomeric RBD subunit	Al(OH)3	92.28%	Severe adverse events were not reported	Aguilar-Guerra et al., 2021; Hernandez-Bernal et al., 2021; Reardon, 2021
NEGVAC/BioE/ Corbevax/Becov2	Biological E. Limited	IM/2	Phase 3 (ongoing)	Recombinant RBD	Al(OH)3 and CpG 1018	NR	NR	Verma, 2021
FINLAY-FR-2/Soberana 02	Instituto Finlay de Vacunas (Cuba)	IM/2	Phase 3 (data reported)	Conjugated vaccine (RBD and TT)	Al(OH)3	71%after two doses and 92.4% after booster dose	Injection site events and fever	Toledo-Romani et al., 2021
UB-612	COVAXX (United States)/United Biomedical Inc. Asia (Taiwan)	IM/2	Phase 2/3 (ongoing)	Multitope peptide based on S1-RBD-protein	Aluminum phosphate	NR	NR	Hasanzadeh et al., 2021
ReCOV	Jiangsu Rec-Biotechnology Co., Ltd.	IM/2	Phase 3 (ongoing)	Recombinant trimeric two-component spike N-terminal domain (NTD) and RBD	BFA03	NR	NR	Biorender, 2021b; Clinicaltrials Gov, 2022
**Nanoparticle vaccines**								
Novavax/NVX-CoV2373	Novavax (United States)	IM/2	Phase 3 (data reported)	Full length recombinant S protein-micelle nanoparticle	Matrix M	89.7% (based on phase 3 in the United Kingdom) and 92.6% (based on phase 3 in the United States and Mexico)	Injection-site tenderness or pain, headache, muscle pain, myalgia, malaise and fatigue (based on phase 3)	Dunkle et al., 2021; Heath et al., 2021
GBP510	SK Bioscience Co., Ltd. (South Korea) and GSK (United Kingdom)	IM/2	Phase 3 (ongoing)	Self-assembled two- component nanoparticle vaccine displaying RBD of spike	AS03	NR	NR	Philippidis, 2021

*NR, not reported; IM, intramuscular; IN, intranasal; Al(OH)3, aluminum hydroxide; TT, tetanus toxoid.*

## Recombinant Spike Protein Vaccines

### VAT00002 Vaccine

Sanofi Pasteur and GSK developed the VAT00002 Sanofi–GSK COVID-19 vaccine, a recombinant protein subunit vaccine containing the SARS-CoV-2 spike protein generated in insect cells using a baculovirus vector. Sanofi provides the recombinant antigen, while GSK (GlaxoSmithKline, Belgium) provides the adjuvant (AS03), which helps the immune system response (Garçon et al., 2012; Goepfert et al., 2021).

To identify a formulation, antigen dose, and a dosing schedule for further development of CoV2 preS dTM vaccine candidate, Sanofi-GSK began Phase 1 studies (VAT00001) in the United States in September 2020 with 439 healthy adults aged 18 and above. This trial showed that Neutralizing and binding antibodies after two doses of vaccine were higher in adjuvanted vs. unadjuvanted groups, in high dose vs. low dose groups, and younger vs. older adults (Pasteur, 2020).

Sanofi-GSK began phase 2 trial with 722 individuals in the United States and Honduras in February 2021. This trial included healthy adults aged 18 and above to assess the immunogenicity, safety, and reactogenicity of two injections given 21 days apart at three antigen dosage levels of 5, 10, and 15 μg (Pasteur, 2021).

The interim results of the phase 2 study showed that the adjuvanted recombinant COVID-19 vaccine candidate produced significant rates of neutralizing antibody responses in all adult age categories, with seroconversion rates ranging from 95 to 100%. High neutralizing antibody levels were also made after a single injection in individuals with signs of previous SARS-CoV-2 infection, indicating that the vaccine has a lot of promise for development as a booster vaccine (Sanofi, 2021b).

Sanofi and GSK began enrolling patients in their Phase 3 clinical study (VAT00008) in May 2021 to assess their adjuvanted recombinant protein’s safety, efficacy, and immunogenicity COVID-19 vaccine candidate. More than 35,000 people aged 18 and above from different countries take part in the global, randomized, double-blind, placebo-controlled Phase 3 study, which takes place in the United States, Asia, Africa, and Latin America. Participants were categorized as naïve (not previously infected) or non-nave (previously infected) based on evidence of the previous disease. The overall number of participants in the study to be about 37,000. The experiment split into two stages. The first stage consists of a monovalent vaccination carrying the original SARS-CoV-2 spike protein (D614). The second stage consists of a bivalent vaccine having both the D614 spike protein and spike protein of the B.1.351 variant (Sanofi, 2021a). This study, NCT04537208, is registered with ClinicalTrials.gov and is currently underway. No findings have been published as of yet since phase 3 is still in progress (Sanofi, 2021c).

In parallel with the phase 3 study, Sanofi Pasteur has launched an extension of the phase 2 booster study (NCT04762680) to evaluate different formulations of the candidate vaccine as a primary series and as a booster dose against COVID-19 in over 4,500 adults who have previously been vaccinated (Sanofi, 2021d). As previously stated, no data from phase 3 and phase 2 boosters was released.

### SCB-2019 Vaccine

Clover Biopharmaceuticals (Chengdu, China) developed SCB-2019, a protein subunit COVID-19 vaccine that combines a trimeric version of the SARS-CoV-2 spike protein (S-Trimer) with one of two adjuvants: AS03 (GlaxoSmithKline) or CpG/Alum (Dynavax) (Liu et al., 2017). The spike protein is presented in its natural three-part form in the vaccine, resulting in a more effective immune response. S-Trimer is a recombinant SARS-CoV-2 fusion protein produced in Chinese hamster ovary cells using Trimer-Tag technology. It maintains S-natural protein’s trimeric structure in the prefusion state of the antigenic epitope, which is required for viral neutralization, and it binds to human ACE2 with great affinity (Liang et al., 2021).

According to pre-clinical studies, S-Trimer adjuvanted with either AS03 or CpG 1018 with alum may elicit potent humoral and cellular immune responses in various animal species and protective immunity against SARS-CoV-2 infection in non-human primates (NHP), with no indications of disease enhancement (Liang et al., 2021).

In June 2020, a Phase 1 randomized, double-blind, placebo-controlled trial clinical research in healthy adult volunteers in Australia with two different adjuvants, AS03 and CpG/Alum, was launched (NCT04405908). Interim findings indicated that the SCB-2019 vaccine, which included S-Trimer protein synthesized with either AS03 or CpG/Alum adjuvants, induced strong humoral and cellular immune responses against SARS-CoV-2, with high virus-neutralizing activity. The above results concluded that both adjuvanted vaccine formulations are well tolerated and suitable for future clinical trials (Richmond P. et al., 2021).

Richmond P. C. et al. (2021) investigated the durability of antibodies in those who participated in phase 1 for up to 6 months after vaccination. SCB-2019 produced dose-dependent immune responses against wild-type SARS-CoV-2 that persisted until Day 184, according to the results of this study. Three of the most frequent variant types of coronavirus, including Alpha (B.1.1.7), Gamma (P.1), and Beta (B.1.351), were cross-reactive with neutralizing antibodies (Richmond P. C. et al., 2021).

Following the positive results of the phase 1 trial, Clover company moved forward with SPECTRA, a global pivotal phase 2/3 clinical trial evaluating the efficacy, safety, and immunogenicity of SCB-2019 (CpG 1018/Alum) in 29,000 adult and elderly participants from five countries across Asia, Latin America, Europe, and Africa (Clover Biopharmaceuticals, 2021a).

Based on Phase 3 results announced by SCB-2019 in September 2021, the studied vaccine showed 67.2% efficacy against all COVID-19 cases of any severity, 84% efficacy against moderate-to-severe COVID-19, and 100% efficacy against severe COVID-19 with hospitalization. Furthermore, vaccine efficacy against the highly transmissible delta form, which is shared worldwide, was 79%. In contrast, vaccine effectiveness against the mu variant, which accounted for one-quarter of strain-identified illnesses, was 59% (Clover Biopharmaceuticals, 2021b).

### COVAX-19 Vaccine

COVAX-19 (or SpikoGen) is a COVID-19 vaccine candidate based on recombinant spike proteins developed by Vaxine Pty Ltd., a South Australian biotech firm. The Advax-SM adjuvant is combined with a recombinant protein antigen in this vaccination. According computer models of the spike protein and human ACE2 receptor that used by Vaxine pty Ltd., it was founded that this vaccine does reduce not only COVID-19 disease but also blocks virus shedding and transmission (Chavda et al., 2021).

In a pre-clinical investigation, the Covax-19 vaccine elicits robust anti-spike antibody and T cell responses in mice. It protects ferrets against SARS-CoV-2 infection when given two consecutive intramuscular doses several weeks apart (Li et al., 2021).

Phase 1 clinical trial for the COVAX-19 vaccine started in June 2020, with 40 healthy adults in Australia randomly allocated to active vaccination (30 participants) or placebo (10 participants). (NCT04453852). Initial findings of this Phase study revealed that the COVAX-19- the vaccine was safe, well-tolerated, and immunogenic in the participants; however, information about the results of this experiment has not been published yet (Khan et al., 2020; Arashkia et al., 2021). COVAX-19 vaccine entered into phases 2 and 3 clinical trials with a cooperation agreement with CinnaGen Company in Iran (Chavda et al., 2021).

In the Phase 2 trial, 400 Iranians participant were allocated to receive two doses of either the active vaccine or saline placebo, 21 days apart, in a 3:1 ratio. This study aimed to assess covax-19′s safety, tolerability, and immunogenicity (Clinical Trials Gov, 2021). CinnaGen claimed that the Covax-19 (SpikoGen) vaccine was well tolerated by all patients and showed a favorable immunogenicity profile (Zamani et al., 2011). However, no results from these studies have been published yet, and this phase is ongoing.

In August 2021, a Phase 3 clinical study of Covax-19 (Spikogen) began in Iran. In this randomized, double-blind, placebo-controlled trial, the efficacy and safety of vaccines will be evaluated in 16,876 adult volunteers. Subjects get two doses of Spikogen vaccine (25 μg) with Advax-SM adjuvant (15 mg) or placebo, 21 days apart, in a 3:1 ratio. Furthermore, due to the prevalence of the Delta variant in Iran, the efficacy of the vaccine against this variant is being assessed as part of this research (Cinnagen, 2021). According to results of clinical studies on this vaccine, CinnaGen reported that Spikogen has produced a robust immune response in 87% of recipients (Khademi et al., 2018). This phase is continuing, and no findings from these trials have been published. On October 2021, Iran has approved the vaccine for emergency use (Peeridogaheh et al., 2013).

### Nanocovax Vaccine

Nanocovax is a protein subunit vaccine produced by Nanogen Pharmaceutical Biotechnology JSC in Vietnam. The vaccine is based on SARS-CoV-2 recombinant spike protein with aluminum hydroxide as adjuvant. Testing the vaccine in animal models has exhibited its capability to create high anti-spike antibody levels. Nanocovax has also been demonstrated to have ability to neutralize anti-spike IgG antibodies and also antibody titers against the Wuhan variant.

The phase 2 trial of the vaccine were conducted at Military Medical Academy and the Pasteur Institute in Hanoi and Ho Chi Minh, respectively. Pain was the local adverse effect observed after the first and the second doses, whereas fatigue and headache were systemic effects found after each injection. However, most AVs were grade 1 and often resolved within 7 days. Hyperglycemia and leukocytosis have been reported, as well. Considering these AVs, none of the phases of vaccination were paused (Jacob et al., 2020). The results of phases 1 and II trials revealed that the vaccine has an excellent safety profile irrespective of the dose. In the phase 3 trial, T-cell responses and vaccine efficiency will be examined (Hosier et al., 2020). The Nanocovax has also been indicated to induce high levels of S protein-specific IgG and neutralize antibody in BALB/c mice, Syrian hamsters, and Macaca leonina. Moreover, the vaccine protected the upper respiratory tract from SARS-CoV-2 infection in a hamster model. The vaccine also showed no adverse effects on Albino Swiss mice, brown rats, and New Zealand rabbits. These pre-clinical results reflected that the Nanocovax vaccine is highly safe, effective, and immunogenic (Deeks et al., 2020).

### Razi Cov Pars

The Iranian-made COVID-19 vaccine, Razi Cov Pars, is a protein subunit vaccine comprising coronavirus-like spike proteins (Kumar and Kumar, 2021). This first injectable inhaled SARS-CoV-2 vaccine was developed by Razi Vaccine and Serum Research Institute (Chaudhary et al., 2021). Its administration into the body is through three doses, two injections and one nasal spray. The injection of the second dose of the vaccine is carried out 21 days later and after 51 days; the third dose will be inhaled later (Tehrantimes, 2021).

The results of its preclinical trials have not been reported yet. Razi’s vaccine was tested in two clinical trials (Ghiasi et al., 2021). The first phase of the study included a double-blind randomized placebo-controlled trial initiated in Iran on 29 January 2021. In this phase, 133 healthy volunteers, with the age range of 18–55, were assigned to four random groups, in which the last group was control.

In the first phase of human clinical test, the vaccine proved to be safe, though mild complications were detected. Phase 2 clinical trial of the vaccine initiated in April 2021 to evaluate the safety and immunogenicity of the vaccine candidate. The same as phase 2, phase 1 was conducted in Iran and included a randomized placebo-controlled trial in which 500 volunteers aged 18–70 participated. Participants were categorized into two study groups comprising of one vaccine group and a placebo group. The first group received a selected vaccine dose from phase 1, and the second group received adjuvant only. The immunogenicity and efficiency of the vaccine indicated in the phase 1 trial were confirmed in the phase 2 (Pilicheva and Boyuklieva, 2021).

### MVC-COV1901 Vaccine

MVC-COV1901 is a recombinant protein subunit vaccine based on the stabilized pre-fusion SARS-CoV-2 spike protein S-2P, adjuvanted with CpG 1018 (an oligodeoxynucleotide which acts as a toll-like receptor 9 agonist) and aluminum hydroxide. The S-2P protein was developed by Wrap and colleagues4 at the US National Institute of Allergy and Infectious Diseases, and the synthetic oligodeoxynucleotide CpG 1018 adjuvant was developed by Dynavax Technologies (Emeryville, CA, United States). The result of a phase 1 trial showed that MVC-COV1901 was well tolerated and elicits robust T-cell and B-cell immune responses. The MVC-COV1901 also indicated an acceptable safety profile and could elicit favorable neutralizing antibody titers. Besides safety, the vaccine was demonstrated to be well tolerated. In young and older adults, it rarely causes febrile reactions. MVC-COV1901 vaccine produces high levels of neutralizing antibodies and induces anti-spike IgG titers, and its seroconversion rate is about 100% by day 57. Considering the WHO IU and BAU conversion models, the clinical efficacy predicted for the vaccine is the same. Phase 2 clinical trial affirmed the progress of MVC-COV1901 in future phase 3 trials. Recently, the vaccine has received authorization for emergency use in Taiwan (Hsieh et al., 2021). In an earlier investigation, the vaccine-induced increased neutralizing antibodies with a 10,000-fold rise in IgG level and a mean of 50-fold higher pseudovirus neutralizing titers in each dose group than vehicle group or adjuvant control. Six days after the infection, vaccinated hamsters showed no weight loss and had considerably decreased lung pathology. Moreover, the viral load levels in the lungs of vaccinated (but not unvaccinated) animals declined to less than detection limit. Vaccination with either 1 or 5 μg of adjuvant S-2P induced similar immunogenicity and protection from infection (Lien et al., 2021).

### EpiVacCorona Vaccine

EpiVacCorona Vaccine (EVCV) is an antigen-based vaccine produced by the Vektor State Research Center of Virology and Biotechnology, Koltsovo, Russia. This vaccine has ability to provoke an immune reaction against COVID-19 and is able to enhance immunity development. The EVCV is dependent on chemically synthesized peptide antigens of SARS-CoV-2 proteins and contains a composition of chemically synthesized peptide immunogens of the S protein of the SARS-CoV-2. The vaccine has been indicated to have ability to conjugate to a carrier protein and adsorb on an adjuvant containing aluminum hydroxide (Ryzhikov A. B. et al., 2021).

Based on evidence, the peptides and the viral part of the chimeric protein need to immunize individuals receiving EVCV against the SARS-CoV-2 and to activate the induction of protective antibodies. The producers of EVCV believe that this vaccine has stability at refrigerator temperatures for up to 2 years. All people who received the vaccine developed specific antibodies against its antigen. However, the efficacy of EVCV is officially uncertain and requires regulatory approval (Abdulla et al., 2021).

### SCTV01C/SCTV01E

The two protein subunit vaccine, called SCTV01C and SCTV01E, has developed by the Chinese company Sinocelltech. The vaccines were injected intra-muscular. CTV01C is a Bivalent SARS-CoV-2 Trimeric Spike Protein Vaccine plus a squalene-based oil-in-water adjuvant used against SARS-CoV-2 variants. 12 trials in 3 phases assessed the safety, tolerability, immunogenicity, and protective effect of the SCTV01C in two countries of China and the United Arab Emirates. In February 2022, SCTV01C entered phase 2 trials as booster shots to assess their ability to generate immunity in adults and adolescents who have already received other vaccines. On 14 September 2021, a phase 2/3 trial, multicenter, randomized, double-blinded trial (NCT05043285) assessed the safety, tolerability, and immunogenicity of SCTV01C in 12,420 participants (18 Years and Older) in the United Arab Emirates that previously vaccinated with inactivated COVID-19. In April 2022, the company conducted phase 3 clinical trial (NCT05323461) to assess the safety of SCTV01C or SCTV01E on 1,800 healthy volunteers (≥18 years old) previously vaccinated with other COVID-19 vaccine or previously diagnosed with COVID-19. SCTV01E is a COVID-19 Alpha/Beta/Delta/Omicron variants S-trimer vaccine used as booster shots in phase 2. On April 4, another phase 3 trial for SCTV01E was conducted on 12,000 healthy volunteers (≥12 years) formerly unvaccinated or fully vaccinated with Sinopharm inactivated COVID-19 vaccine or mRNA/adenovirus vectored vaccine. This vaccine is not yet approved (Keech et al., 2020).

## Recombinant RBD Vaccines

### ZF2001 Vaccine

ZF2001 is an adjuvanted protein subunit vaccine developed by Anhui Zhifei Longcom Biologic Pharmacy Co and the Institute of Medical Biology at the Chinese Academy of Medical Sciences that utilizes a tandem-repeat SARS-CoV-2 spike receptor-binding domain (RBD) dimer as the antigen. To better immunogenicity, the protein was designed as a tandem-repeat dimeric RBD (Premkumar et al., 2020; Wang N. et al., 2020; Wang Q. et al., 2020). The RBD-Dimer model was created using clinical-grade Chinese hamster ovary (CHO) cell lines, and then combined with aluminum hydroxide as an adjuvant to make the final vaccine (Pourmalek et al., 2021).

Dai et al. (2020) described the structure-guided design of a coronavirus immunogen composed of two protein subunits containing the virus spike RBD. They joined together through a disulfide bond or tandem repeat. Their results demonstrated that the compared to the conventional monomeric form of SARS-CoV-2 RBD, the dimeric form of RBD increased immunogenicity in a mouse model (Dai et al., 2020).

Yang et al. (2021) performed two randomized, double-blind, placebo-controlled, phase 1 and phase 2 trials in China. Participants in the phase 1 study were randomly allocated (2:2:1) to receive three intramuscular doses of the vaccine (25 or 50 μg) or a placebo 30 days apart. In phase 2, individuals were randomized (1:1:1:1) to receive the vaccination (25 or 50 μg) or placebo intramuscularly in two or three doses 30 days apart. According to the findings of Phase 1 and 2 studies, 83% of individuals developed neutralizing antibodies after two doses of the vaccine, and 97% developed neutralizing antibodies after three doses. Furthermore, none of the participants had any severe adverse effects. The 25 μg/three-dose group had the most excellent SARS-CoV-2 neutralizing response (Yang et al., 2021).

NCT04646590 is a randomized phase 3 study that is presently ongoing. It was first registered in November 2020, with plans to enroll 29,000 healthy adults 18 years and older in China, including 750 participants aged 18–59 years and 250 participants aged 60 years and over; 21,000 participants aged 18–59 years and 7,000 participants aged 60 years and over will receive 25 μg of the vaccine over a 0, 1, and 2-month schedule. According to early phase 3 findings released by Zhifei, the vaccines effectiveness rate against COVID-19 patients of any severity and delta variant was 81.76 and 77.54%, respectively. Although preliminary findings have not been peer-reviewed, this vaccine has been approved for emergency use in China and Uzbekistan since March 2021 (The Straits Times, 2021).

Huang et al. (2021) evaluated neutralization activity in 24 serum samples from subjects in two clinical trials, 12 of which were vaccinated with BBIBP-CorV (inactivated vaccine). The others were immunized with ZF2001 against variant 501Y.V2 and SARS-CoV-2 wild type. Based on their results, the variant 501Y.V2 did not escape immunity evoked by vaccines target the whole virus (BBIBP-CorV) or S protein dimeric RBD vaccines (ZF2001) (Huang et al., 2021).

Zhao et al. (2021) tested the neutralization efficacy of ZF2001 against coronavirus variants using a pseudotyped virus expressing SARS-CoV-2 spike. They discovered that the ZF2001 vaccination maintained its neutralizing effectiveness against the newly discovered delta variant. However, this vaccination demonstrated a more significant decrease in activity against the beta variant. Furthermore, individuals with a longer gap between the second and third doses (doses at 0, 1, and 4–6 months) had more significant neutralizing action (Zhao et al., 2021).

In a study by Cao et al. (2021), the humoral immune response to circulating SARS-CoV-2 variants such as 501Y.V2 (B.1.351) in plasma and neutralizing antibodies elicited by CoronaVac (inactivated vaccine), ZF2001 (RBD-subunit vaccine), and natural infection, was investigated. Their findings revealed that RBD-subunit vaccines, such as ZF2001, had much greater tolerance to 501Y.V2 than convalescents. Furthermore, a longer interval between the third and second doses of ZF2001 results in more robust 501Y.V2 neutralizing activity than the usual three-dose administration. These researchers found that launching RBD vaccines through a third-dose boost may be suitable for fighting SARS-CoV-2 strains (Cao et al., 2021).

### Abdala (CIGB-66) Vaccine

Abdala, with the technical name CIGB-66, is a Cuban vaccine developed by the Center for Genetic Engineering and Biotechnology (CIGB) and is based on the recombinant RBD subunit of the spike protein of the SARS-CoV-2 virus. The vaccine was produced in Pichia pastoris yeast containing aluminum hydroxide as adjuvant. The phase 1/2 clinical trial of the vaccine was initiated on December 7, 2020, and the result indicated that the vaccine does not need a carrier protein to acquire high levels of this indicator. The phase 3 trial began on March 22, 2021 and showed 92.28% efficacy. The vaccine was affirmed for emergency use on July 9. The Abdala vaccine aims to introduce antibodies interfering with the entry of the pathogen into cells, a fundamental mechanism create protection.

CIGB was selected Pichia pastoris as an expression system because of its experience in using the technological platform, which is cheaper than other platforms. As RBD is a covalently associated glycoprotein, the saccharides of *P. pastoris* provide this domain with an immunopotentiating impact that contributes to immunogenicity. Abdala was designed by means of protein engineering using structural bioinformatics computational methods aimed at increasing its similarity to the SARS-CoV-2 virus (Shalash et al., 2021). Recently, it has been reported that the Abdala vaccine has more than 90% effectiveness against severity and death, notwithstanding the prevalence of the Delta variant of SARS-CoV-2 (Lemos-Perez et al., 2021). In July 2021, Abdala commenced clinical trial phase I/II for children and adolescents aged 3–18.

### BECOV2

The COVID-19 vaccine candidate BECOV2, also known as Corbevax or NEGVAC or BioE COVID-19, was developed by Indian company Biological E. (located in Hyderabad) in collaboration with a group including Baylor College of Medicine [United States; Texas Children’s Hospital (Center for Vaccine Development)] and Dynavax Technologies Corporation (United States) (Dilipkumar, 2021; Verma, 2021). This vaccine was approved in India and Botswana but for emergency use. This vaccine is based on a recombinant protein subunit of spike protein (construct of RBD N1C1 made by Baylor College of Medicine) in a combination of alum adjuvant with Dynavax Technologies Corporation’s CpG (made by Dynavax) that elicited a robust immune response against coronavirus (Hodgson et al., 2021). In phase 1/2 clinical trial (CTRI/2020/11/029032), the vaccine was administered intramuscularly in a two-dose schedule (0.5 ml; day 0 and day 28) to select a better vaccine formulation based on overall safety and immunogenicity observation. Following reported promising results in phase 1/2 clinical trials, BECOV2 has completed two phases 2/3 clinical trials. After establishing the safety and immunogenicity of the vaccine in 1,268 COVID-19-negative adult subjects (18–80 years) at two doses with 28-day intervals in the first phase 2/3 clinical trial (CTRI/2021/06/034014), the vaccine is further investigated in second phase 2/3 clinical trial (CTRI/2021/10/037066) on 624 children and adolescents aged between < 18 years and ≥ 5 years with a booster dose on Day 208. In this phase, the vaccine induces a robust immune response without any severe AEs among the pediatric population as young as 5 years old. Currently, the vaccine is approved for children between 5 and 12 but is restricted to use in emergency situations. In phase 3 (CTRI/2021/08/036074), the BECOV2 vaccine was compared with Covishield on more than 2,140 COVID-19-negative adult subjects (18–80 years) at 33 study sites across India. This study demonstrates that the BECOV2 vaccine induces a superior immune response than the Covishield vaccine. Also, it had 50% fewer adverse events than Covishield. A researcher showed that this vaccine’s effectiveness is 90% against the Ancestral-Wuhan strain and 80% against the Delta strain to prevent symptomatic infections (Speiser and Bachmann, 2020).

### FINLAY-FR-2 Vaccine

The Finlay Vaccine Institute in Cuba developed the FINLAY-FR-2 (Soberana 02) COVID-19 vaccine. It is a conjugate vaccine, which means that the viral antigen, the RBD, is chemically coupled to the tetanus toxoid to keep it stable. Aluminum hydroxide is used as an adjuvant in Soberana 2 to strengthen the immune (Valdes-Balbin et al., 2021).

Valdes-Balbin et al. (2021) demonstrated in pre-clinical investigations that macromolecular constructs containing recombinant RBD coupled to tetanus toxoid elicit a robust immunological response in laboratory animals.

According to the Cuban Public Registry of Clinical Trials, a phase 1 clinical trial with 40 volunteers began in October 2020, with an open, sequential, and adaptive study to evaluate the vaccine’s safety, reactogenicity, and immunogenicity (Soberana 02, 2021).

Following early findings, the vaccine based on RBD6-TT/alum was advanced to a phase 2 clinical study in December 2020. Phase 2a of the vaccination included 100 Cubans, while Phase 2b included 810 volunteers aged 19–80 years old (Universo Online, 2021). Initial studies indicated that the vaccination elicited an immunological response after 14 days (OnCubaNews English, 2020). Phase 3 commenced at the beginning of March as initially scheduled. The 44,010 volunteers were divided into three groups: some received two doses of the vaccine 28 days apart; another group will get two doses plus a third immune booster (Soberana Plus), and the third a placebo (ABS CBN, 2021).

Pasteur Institute of Iran performed Phase 3 on 24,000 people aged 18–80 years old in 8 cities as part of the collaboration with other nations to develop the COVID-19 vaccine (IRCT, 2021). Although clinical study results have not yet been published, preliminary reports suggest a 62% efficacy after just two doses (Acosta, 2021). When used in conjunction with a Plus booster dose, the vaccination was also 91.2% effective, according to BioCubaFarma (The Indian Express, 2021).

### UB-612 Vaccine

The UB-612, the first COVID-19 “multi-tope” protein-peptide vaccine, was produced by United Biomedical Inc. Asia in Taipei. The vaccine comprises of eight components and includes a strong S1-RBD component connected to a single-chain fragment region (sFC) of a human IgG1. This region, which functions as the principal neutralizing domain of the virus, facilitates cell attachment. The UB-612 vaccine consists of a proprietary peptide UBITh1a^®^, five peptides with Th or CTL epitopes, and aluminum phosphate adjuvant. With the aim of inducing a broad immune response, the vaccine was designed and was able to mitigate viral load and block COVID-19 infection in mice and monkeys. In rats, UB-612 toxicity has been displayed to have appropriate safety (Chaudhary et al., 2021; Hasanzadeh et al., 2021).

In Guirakhoo et al.’s (2020) study, UB-612 vaccine was composed of eight components, six synthetic peptides, a proprietary CpG TLR-9 agonist, and aluminum phosphate adjuvant. The phase 1 trial and additional trials conducted globally have denoted that the vaccine is highly promising and a differentiated candidate for inhibiting SARS-CoV-2 transmission and COVID-19 disease.

According to the pre-clinical investigations in guinea pigs, rats, and mice, the UB-612 vaccine could induce extremely high titers of neutralizing antibodies with S1-RBD:hACE2 inhibition activities and could generate balanced Th1/Th2 response toward the Th1 polarity. The vaccine also could induce balanced B cell activation and enhance T cell immune response in human body, hence providing exceptional protection. Moreover, the UB-612 vaccine could generate a favorable immune response in humans and is well tolerated without serious AVs. The vaccine does not need to be transported and stored in an ultra-low temperature. This feature is speculated to be more competitive than other RNA vaccines produced by companies such as Pfizer/BNT (Kanno et al., 2021).

### ReCOV

China’s Jiangsu Rec-Biotechnology developed the ReCOV vaccine. This recombinant protein two-component COVID-19 vaccine is grown in Chinese hamster ovary cells. This vaccine consists of the RBD of the spike protein (protein engineering platforms) and a novel adjuvant, which enters the host cells by binding to angiotensin-converting enzyme 2. After vaccination, the host will induce neutralizing antibodies against the spike protein to protect them from infection with the native virus (Biorender, 2021a). On March 26, a phase 1 clinical trial was launched on 160 healthy volunteers from 18 to 80 years, at two doses with 21 days interval, to evaluate the safety, reactogenicity, and immunogenicity in New Zealand. Following vaccine effective at generating immunity, this company incorporated Shenzhen Rhegen Biotechnology and founded the company of Wuhan Rhecogen Biotechnology on September 29. In late 2021, this new company conducted a Phase 2/3 trial to evaluate the efficacy, safety, and immunogenicity of ReCOV in 18–80 years adult volunteers in the Philippines. The data results showed that ReCOV induced high levels of neutralizing antibodies against variants of coronavirus without side effects. In April 2022, it received approval to extend the trial to the U.A.E. ReCOV has not yet been approved (Ghiasi et al., 2021).

## Nanoparticle Vaccines

### NVX-CoV2373 Vaccine

The NVX-CoV2373 protein subunit vaccine developed by Novavax contains Matrix-M1 adjuvant2 and a recombinant SARS-CoV-2 (rSARS-CoV-2) nanoparticle vaccine 3 from the full-length, wild-type SARS-CoV-2 spike glycoprotein. It targets antibody and vaccine development because it enhances viral attachment to the host cell’s human angiotensin-converting enzyme 2 (hACE2) receptor. Using the saponin-based matrix M1 adjuvant, the lack of cell-mediated immune responses, which characterize protein subunit vaccinations, was overcome (Du et al., 2009; Bengtsson et al., 2016; Keech et al., 2020; Tai et al., 2020). Matrix M-adjuvanted NVX-CoV2373 was evaluated for immunogenicity in animal models such as rats and baboons. In pre-clinical studies, NVX-CoV2373 generated antibodies that blocked the binding of spike protein to cellular receptors and protected against infection (Mandolesi et al., 2020; Tian et al., 2021).

Following early encouraging findings in animal models, Novavax launched a Phase 1/2 trial. Clinical results from Novavax’s phase 1 and 2 trials revealed that a two-dose regimen given 21 days apart was safe and generated more significant levels of coronavirus antibodies than those observed in COVID-19 survivors. The vaccination also increased T cells, another component of the human immune system (Keech et al., 2020).

Novavax developed a Randomized, Observer-Blinded, Placebo-Controlled Study phase 2 a/b study in South Africa to evaluate the effectiveness, safety, and immunogenicity of the Novavax vaccine in adults subjects living without HIV and adults living with HIV (ClinicalTrials.gov NCT04533399). At the time of this Phase, B.1.351 variant was operating throughout the country and beyond. An early analysis of this clinical trial data revealed that total vaccination effectiveness was 49%. Among HIV-negative subjects, Novavax was 60% effective. Furthermore, vaccine effectiveness against the B.1.351 variant was 51.0% (Shinde et al., 2021). Severe and medically attended adverse effects were uncommon. Consequently, the findings of this clinical trial demonstrated that the NVX-CoV2373 vaccination had greater effectiveness in preventing Covid-19 among HIV-negative individuals (Callaway and Mallapaty, 2021). A phase 3 randomized, observer-blinded, placebo-controlled study was performed at 33 locations in the United Kingdom between September and November 2020, recruiting up to 9,000 individuals to evaluate the Novavax’s effectiveness, safety, and immunogenicity (NCT04583995). The participants’ ages varied from 18 to 84. Two doses of Novavax vaccine given 21 days apart in adults offered 89.7% protection against symptomatic Covid-19 caused by both B.1.1.7 and non-B.1.1.7 variants, according to the results (overall efficacy). This vaccination was 86.3% effective against B.1.1.7 (or alpha) variants and 96.4% effective against non-B.1.1.7 variants. In most instances, reactogenicity was minor and transient. The occurrence of significant adverse events (AVs) was minimal and equivalent in both groups (Heath et al., 2021).

PREVENT-19 trial NCT04611802 is a randomized phase 3 study, including 29,960 adult volunteers that performed in the United States and Mexico (Shirmohammadi-Khorram et al., 2019). The participants were entirely (100%) immune to both mild and severe diseases. The vaccine was 90.0% effective in avoiding symptomatic COVID-19 illness in individuals at high risk of acquiring COVID-19 issues. According to safety data, the experimental vaccine was usually well-tolerated (Johnson, 2021).

### GBP510 Vaccine

The GBP510 (recently known as SKYCovione) is a self-assembled nanoparticle vaccine developed by the South Korean company SK Bioscience and the Institute for Protein Design (IPD) at the University of Washington with a combination of GlaxoSmithKline’s (GSK; a British multinational pharmaceutical company) adjuvant (AS03). GBP510 is a recombinant protein-based vaccine that targets the RBD of the SARS-CoV-2 spike protein (Smith et al., 2020).

The phase I/II results demonstrate a high level of neutralizing antibody with a 100% seroconversion rate in healthy adults (aged 19–85 years) given the adjuvanted vaccine. The phase III trial (NCT05007951) of the vaccine was started with 4,037 participants (570 participants in Korea and 3,467 other countries) over 18-year-old in Thailand, Vietnam, New Zealand, Ukraine, the Philippines, and South Korea in cooperation with 16 institutions. The results show this vaccine only elicited a superior neutralizing antibody (over 95%) than AstraZeneca’s Covid-19 vaccine (79%, control vaccine), Vaxzevria, in subjects aged > 65 but induced 2.93 times notarization antibody titer 2 weeks after of the second dose that of a control vaccine. Also, this vaccine showed a favorable safety profile compared to the control vaccine (mild or moderate adverse reactions) in this vaccine. This vaccine is not yet approved (PMLiVE, 2021).

## Conclusion and Outlook

Producing effective vaccines based on Protein Subunit against SARS-CoV-2 Infection as rather new technology has become a use full choice. Here, we reviewed 16 vaccines passing phases 3 and 4 from different countries, Australia, the United States, India, Russia, China, South Korea, and Iran. SARS-CoV-2 spike protein S is a large, trimeric glycoprotein that plays the most important roles in viral attachment, fusion and entry, and serves as a target for the development of antibodies, entry inhibitors and vaccines. In these vaccines, different protein particles such as full-length wild-type SARS-CoV-2 spike glycoprotein, SARS-CoV-2 spike protein (D614), tandem-repeat SARS-CoV-2 spike receptor-binding domain (RBD) dimer. The utilization of aluminum hydroxide as adjuvant was common in most vaccines. In addition, CpG 1018 in MVC-COV1901 and Matrix-M1 in NVX-CoV2373 were some other adjuvants.

Overall, the occurrence of adverse events in all reviewed Protein Subunit Vaccines was uncommon or minimal. In Iran COVAX-19, Razi Cov Pars and Soberana-02 are three Protein Subunit Vaccines that are approved for use. From an administration way point of view, except Razi Cov Pars that uses two injections and one nasal spray dose, other vaccines have two or three intramuscular doses. Vaccines with intranasal spray formulation can be easily administered and would be beneficial as mucosal immunity is known as the first line of defense against the virus. Early protection against COVID-19 infection is related to the secretion of mucosal IgA in the upper respiratory tract during initial contact with the SARS-CoV-2 virus. In conclusion, despite all efforts to produce effective vaccines, the appearance of new mutated SARS-CoV-2 strains is a serious challenge. Present vaccine proficiency must be evaluated and their efficiency reported.

Based on available documentation from clinical trials, five of the protein subunit vaccines have reported the efficacy which is as follows: Abdala (92.28%), SOBERANA 02 Plus (92.4%), Novavax (92.6%), EPIVACCORONA (79%), and SCB-2019 (67.2%). Among these, Abdala, SOBERANA 02 Plus, and Novavax have shown high efficacy and safety profile, but more precise, the results of Phase III trials of Novavax, SOBERANA 02 Plus, and SCB-2019 have published by more detailed analyses. In addition to favorable results of these candidated vaccines, in our view, Novavax may be one of the promising vaccines because it could elicits protective immunity against broad spectrum of variants.

This vaccine contains the spike protein of the coronavirus itself, but formulated as a nanoparticle, which cannot cause disease. It is simpler to make than some of the other vaccines and can be stored in a refrigerator (2–8°C), making it easier to distribute. Novavax is shown to be highly effective in clinical trials. Ninety percent effective against lab-confirmed, symptomatic infection and 100% against moderate and severe disease in Phase 3 trial results released. In a Phase 3 study conducted in the United States and Mexico during a period in which multiple variants (Alpha, Beta, and Delta) were in circulation, vaccine efficacy against mild, moderate, or severe COVID-19 was 90%.

Based on clinical trial conducted in the United Kingdom, the efficacy was 86.3% against the B.1.1.7 (alpha) variant and 96.4% against non-B.1.1.7 variants. On the other hand, based on clinical trial performed in the United States and Mexico, efficacy against the alpha variant was 93.6% and against any variant was 92.6%. Moreover, the efficacy against the latter variants was reported 100%. Novavax says its vaccine can generate an immune response against Omicron, but scientists are still learning about this. Prevention of severe disease and mortality is the main objective of vaccination. Hence, the occurrence of serious adverse effect was low and transient, and also no hospitalizations or death was occurred among the vaccinated recipients.

## Author Contributions

MH and SK contributed to the conception, design of the work, and acquisition. VK, MS, RG, MM, MS, HG, MH, and SK contributed in drafting of the manuscript, revising and final approval of the version to be published. All authors contributed to the article and approved the submitted version.

## Conflict of Interest

The authors declare that the research was conducted in the absence of any commercial or financial relationships that could be construed as a potential conflict of interest.

## Publisher’s Note

All claims expressed in this article are solely those of the authors and do not necessarily represent those of their affiliated organizations, or those of the publisher, the editors and the reviewers. Any product that may be evaluated in this article, or claim that may be made by its manufacturer, is not guaranteed or endorsed by the publisher.
